# Surgical Management of the Peripheral Osteoma of the Zygomatic Arch: A Case Report and Literature Review

**DOI:** 10.1155/2019/6370816

**Published:** 2019-06-18

**Authors:** Umberto Autorino, Claudia Borbon, Maria Chiara Malandrino, Giovanni Gerbino, Fabio Roccia

**Affiliations:** Division of Maxillofacial Surgery, Surgical Science Department, Città della Salute e delle Scienza Hospital, University of Turin, Corso A.M. Dogliotti 14, 10126 Torino, Italy

## Abstract

An osteoma is a benign, slow-growing, osteogenic neoplasm with a low recurrence rate that is typically characterized by the proliferation of a compact or cancellous bone. It can be peripheral, central, or extraskeletal. Usually asymptomatic, peripheral osteomas in the maxillofacial region commonly arise in the paranasal sinuses and mandible and rarely occur in the zygomatic arch, with only six previously documented cases in the literature. Here, we present the management of a solitary peripheral osteoma of the right zygomatic arch in a 72-year-old woman and a review of the literature.

## 1. Introduction

Osteoma is a benign, slow-growing, osteogenic neoplasm characterized by the proliferation of a compact or cancellous bone [1]. It was first recognized as a tumor by Jaffe [2]. The etiology of these lesions remains unknown, but several explanations have been suggested for its origin including embryologic, traumatic, inflammatory, metaplastic, and genetic causes [1, 3–5]. It is more common in females, with a mean age at diagnosis of about 50 years [4, 5]. They can occur as solitary or multiple lesions, the latter of which can be seen in cases of Gardner syndrome [6].

Osteomas almost exclusively affect the maxillofacial skeleton, particularly the paranasal sinuses and mandible, and can be classified based on the location from which they arise. Central osteomas arise centripetally from the endosteum, peripheral osteomas are caused by centrifugal growth of the periosteum, and extraskeletal soft tissue osteomas develop within the muscles [1, 4, 5, 7]. The majority of these lesions are peripheral varying from 41.9% [4] to 49% [1], while central osteomas in the craniofacial skeleton are uncommon. Peripheral osteomas of the craniofacial region occur in the frontal, ethmoid, and maxillary sinuses with no sex or age predilection [3, 8–12].

Clinically, peripheral lesions appear as unilateral, pedunculated, and asymptomatic mushroom-like masses and can produce pain, trismus (when there is nerve involvement), limited mandibular movement, malocclusion, swelling, and facial asymmetry [3, 8, 10–13].

Computer tomography (CT) is the current gold standard for the diagnosis and surgical management of an osteoma. A peripheral osteoma is seen as an oval, radiopaque, well-circumscribed mass attached to the cortex by a broad base or pedicle [3, 10, 13, 14]. Differential diagnosis should include fibrous dysplasia, exostosis, chondroma, ossifying fibroma, condensing osteitis, osteoblastoma, Paget's disease of the bone, osteosarcoma, or odontoma if the lesion occurs near the teeth [15]. Histologic classification can differentiate between two types of peripheral osteomas: compact osteomas, which are composed of mature lamellar bone that do not contain any fibrous component, and trabecular osteomas composed of cancellous trabecular bone and bone marrow surrounded by a cortical margin [3, 10, 13]. Osteomas have a low recurrence rate when treated using adequate surgical techniques. Although excision is recommended for growing or symptomatic lesions, there have been no reports of malignant transformation in the literature [1, 3–5, 10, 13].

We describe a rare case of peripheral osteoma in the zygomatic arch and present a review of the literature.

## 2. Case Report

**Figure 1:**
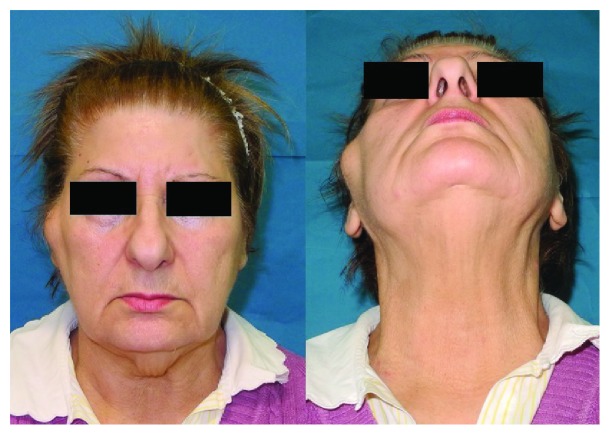
Clinical preoperative view showing preauricolar swelling on the right side of the face.

**Figure 2:**
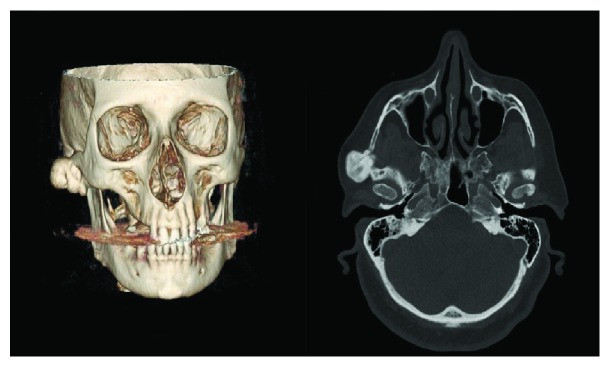
Axial CT scan and three-dimensional reconstruction image showing a well-defined, pedunculated, and radiopaque mass of the right zygomatic arch.

A 72-year-old woman was referred to the division of Maxillofacial Surgery, Città della Salute e della Scienza Hospital, University of Turin (Torino, Italy), for an enlarged preauricular mass on the right side of her face. The lesion had slowly been growing for 3 years (Figure 1). There was no previous history of facial trauma. Her medical history was only remarkable for arterial hypertension and diabetes mellitus type II. Examination revealed a solitary, smooth, nontender, firm, bony asymptomatic swelling over the right zygomatic arch measuring approximately 3 cm in diameter. There were no recent changes in her ability to open her mouth, and no abnormalities were noted in either temporomandibular joint. CT scans confirmed the presence of a 3 cm pedunculated, well-circumscribed, radiopaque, lobulated structure along the lateral border of the right zygomatic arch (Figure 2). Based on the clinical and radiographic findings, we diagnosed a peripheral osteoma of the zygomatic arch. Given the ongoing growth and cosmetic concerns, the decision was made to surgically remove the tumor.

Under general anaesthesia, the zygomatic arch was accessed via a preauricular incision with temporal extension (Al-Kayat and Bramley's modifications [16]). After incision, a complete view of the lesion was obtained and the tumor was easily excised. Histology revealed that the specimen was a normal cortical trabecular bone, confirming the diagnosis of an osteoma.

**Figure 3:**
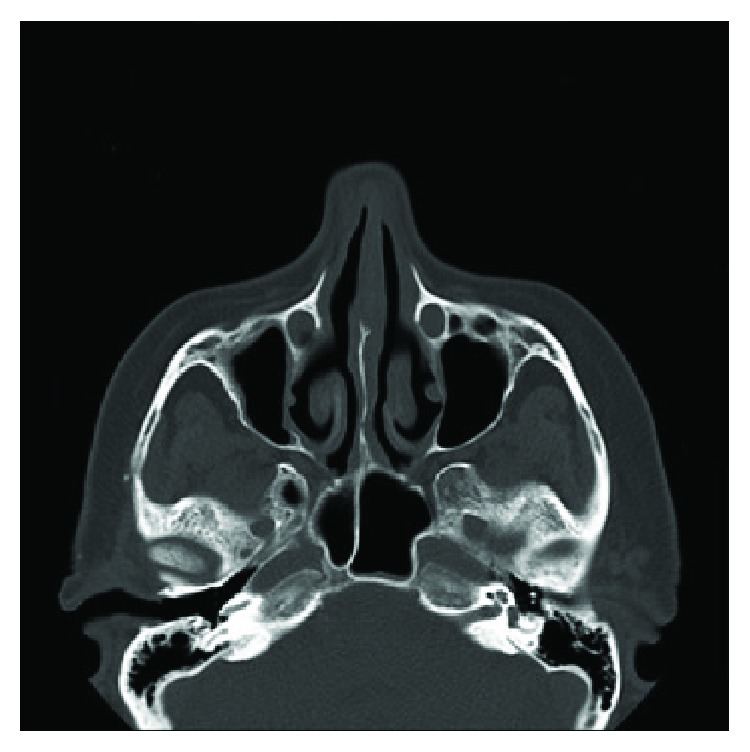
Postoperative axial CT scan.

**Figure 4:**
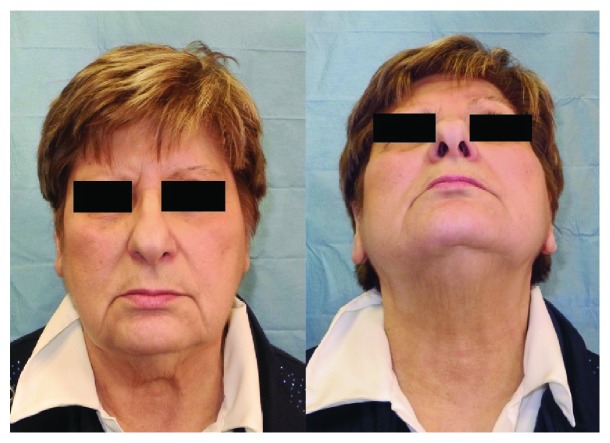
Clinical view 1 year after surgery.

The patient's postoperative course was uneventful with only temporary dysesthesia along the V3 branch of the trigeminal nerve. The symptoms resolved spontaneously after 2 months. The patient was discharged home 5 days after surgery. CT scans 1 year after surgery showed normal bone architecture of the right zygoma, good symmetry, and no signs of relapse (Figures 3 and 4). No clinical evidence of recurrence was encountered at the 5-year follow-up.

## 3. Discussion

Peripheral osteomas of the maxillofacial region most frequently occur in the paranasal sinuses but can also be found in the jaw bones, external auditory canal, orbit, temporal bone, and pterygoid processes [3, 10, 13, 17]. Peripheral osteomas of the zygomatic arch are extremely uncommon. A literature review identified only six previously documented cases [18–23] (Table 1). According to Longo et al. [11] and Kashima et al. [14], the age of patients ranges from 20 to 72 years with a mean age of 47 years. In our study, despite the small number of cases, the prevalence of peripheral osteomas was slightly higher in women.

Several authors [1, 3, 10, 13] have reported that peripheral osteomas are usually asymptomatic. When present, the most common symptoms are pain, trismus, or limited mouth opening. In our review, we found that four of seven patients complained of pain or local dysesthesia, while the remainder of patients were asymptomatic. There did not appear to be any significant correlation between severity of the clinical symptoms and tumor size. Any clinical effects related to the growth of the osteoma were likely due to mass effects of the lesion and compression of adjacent anatomical structures [19, 20, 22, 23]. Surgery is indicated when a patient complains of symptoms or if a lesion presents as progressive growth, as was the case with our patient. An intraoral approach is preferable to avoid facial scarring and facial nerve damage. However, in our review, we found that all lesions were excised through an extraoral approach. A preauricolar approach was used in five cases, and one other report utilized a direct approach by “following the facial skin crease” [19].

Similar to Furlaneto et al. [18], we believe that a preauricular approach allows for adequate surgical exposure, a good view of the lesion, and complete tumor resection. Moreover, this approach has lower risks of facial injury and scarring than direct approaches. Akinmoladun et al. [19] reported a case in which the “tumor was excised under local anaesthesia by direct facial approach using an incision following the facial skin crease,” but “caution was taken to avoid possible damage to the zygomatic arch.” Furlaneto et al. [18] also described a case of osteoma recurrence in the zygomatic arch 10 years after surgical removal using a direct approach. In this case, it is likely that underexposure and poor visibility led to incomplete removal of the tumor.

Most cases of tumor excision have been performed with zygomatic arch preservation, with the exception of a case reported by Quintans et al. [22] , in which complete tumor excision required osteotomy of the zygomatic arch. In this case, positioning of a titanium plate was necessary to restore the normal bone architecture.

## Figures and Tables

**Table 1:** Summery of the published cases of peripheral osteomas of the zygomatic arch.

Case	Age/sex	Clinical symptoms	Size (cm)	Surgical approach	Follow-up	
1	55/F	None	Not reported	Preauricolar	Not reported	Furlaneto et al.
2	20/M	Local sensitivity	3 × 4	Direct facial approach	6 months	Akinmoladun et al.
3	61/M	None	8.5 × 6.7 × 9.85	None	Not reported	Durao et al.
4	35/M	Pain	2	Not reported	Not reported	Naikmasur et al.
5	32/F	Pain	3	Preauricolar	7 years	Quintans et al.
6	55/F	Pain	1.2 × 1.4	Preauricolar	1 year	Starch-Jensen
7	72/F	None	2.7 × 2 × 2.2	Preauricolar	5 years	Present case
